# An exploration of problem posing-based activities as an assessment tool and as an instructional strategy

**DOI:** 10.1007/s41039-015-0006-0

**Published:** 2015-06-23

**Authors:** Shitanshu Mishra, Sridhar Iyer

**Affiliations:** 1 Inter-disciplinary Program in Educational Technology, Indian Institute of Technology Bombay, Powai, Mumbai 400076 India; 2 Department of Computer Science and Engineering, Indian Institute of Technology Bombay, Powai, Mumbai 400076 India

**Keywords:** Problem posing, Instructional strategy, Assessment tool, Knowledge unfolding, CS1, Computer Science Application courses

## Abstract

**Background:**

Problem posing, the generation of questions by learners, has been shown to be an effective instructional strategy for teaching–learning of complex materials in domains such as mathematics. In this paper, we demonstrate the potential of problem posing in two dimensions. Firstly, we present how problem posing can result in unfolding of knowledge and hence how it can be used as an instructional strategy. Then we present another problem posing-based activity as an assessment tool in an Introductory Programming course (CS1).

**Method:**

To explore the potential of problem posing as an instructional strategy, we conducted field studies in the two CS application courses (Data Structures (DS) and Artificial Intelligence (AI)), in which we provided a semi-structured problem posing situation to students. We performed inductive qualitative research and development the questions generated by students using grounded theory-based qualitative data analysis technique. To explore the potential of problem posing as an assessment tool, we conducted a field study in CS1 wherein we employed another problem posing (PP)-based activity in a large class for assessing the learning of computational thinking concepts in an introductory programming course and analysed how performance in traditional assessment tools (quiz score) is related with performance in our non-traditional assessment tool (quality of problems posed during a problem posing activity).

**Results:**

From the studies in DS and AI courses we found that students pose questions and unfold knowledge using seven strategies — Apply, Organize, Probe, Compare, Connect, Vary, and Implement. From the field study performed in the CS1 course we found that the quality of the problems posed (difficulty level) were mostly aligned to the traditional assessment results in the case of novice learners but not in the case of advanced learners.

## Background

Problem posing (PP) refers to the generation of a new problem or a question by learners based on the given situation (Mishra and Iyer 2013). PP has been shown to be useful for identifying knowledge deficit, and opens a way to knowledge exploration. Stoyanova & Ellerton (1996) describe three problem posing situations: free situation, structured situation, and semi-structured situation. Different situations result in the different quality of questions. Variations on these situations can be used to design various PP based activities for different purposes. In this paper we have explored the potential of PP in two dimensions, viz., PP as an instructional strategy, and PP as an assessment tool.

### Overview

In the first part of this paper, we describe a PP based instructional strategy and report its effect on students’ exploration based learning. We find that the PP based instructional strategy proposed in this research is a way to enable exploration based learning, where students unfold knowledge and explore the course content by posing problems. This exploration based learning inculcates a feeling of ownership of the learning process in the students. The students’ open feedback show that students enjoyed the PP based instruction more than the traditional instructions.

In the second part we describe a field study conducted in CS1 course to explore the assessment potential of PP. We found that students were able to demonstrate their learning through question generation and PP can be used as an assessment tool. We find that all possible computational thinking concepts (Brennan and Resnick 2012) were demonstrated by students generated questions. We also find that different qualitative aspects of the questions help in determining different set of assessment objectives.

In the next section we present motivation and a summary of related studies in the domain of problem posing. Further two sections detail the two explorations, i.e., exploring the potential of PP as an instructional Strategy and as an assessment tool respectively. The last section contains the discussion and conclusion of this research.

## Motivation and related study

In this section, we report the related research showing how PP has been explored by education researchers.

### Motivation

Problem posing education is a term coined by Brazilian educator Paulo Freire in his 1970 book “Pedagogy of the Oppressed”. Freire defines this term as a method of teaching that emphasizes critical thinking for the purpose of liberation (Wallerstein 1987). Freire used a problem posing educational model as an alternative to traditional instructionist approach. Human problem posing is extremely limited in both quantity and quality (Graesser et al. 2008). Except for few skilled learners, most human learners lack the essential skill of intelligent inquiry that they can use to enhance their learning. There are very few people who know their knowledge deficit (Hacker et al. 1998). Most people pose very few and shallow problems (Dillon 1990; Good et al. 1987; Graesser and Person 1994).

“A typical student in a class asks less than 0.2 questions per hour in a classroom and the poverty of classroom questions is a general phenomenon across cultures” (Graesser and Person 1994). In addition to the quantity of questions that learners/tutors ask, the quality of questions posed also affects learning (Scardamalia and Bereiter 1992; Graesser et al. 2005; Vanderwende 2008). As compared to deep questions (for example why, why not, how, what-if, what-if-not), shallow questions (who, what, when, where) are asked more by learners and teachers. Generation of both shallow- and deep-level questions is highly important in any teaching–learning environment. Researchers in cognitive science and education recommend teaching–learning environments that encourage students to pose more and good quality questions (Graesser et al. 2008; Beck 1997; Edelson et al. 1999). Explicit training for PP is essential for students and even for instructors.

Limitations in problem posing capability are found in other situations, such as teachers in classrooms asking shallow questions over 90 % of the time (Dillon 1990) and tutors find it difficult to generate good hints and prompts to get students engaged in productive learning trajectories (Chi et al. 2001; Corbett and Mostow 2008; DiPaolo et al. 2004). Tutors also need to pose good questions to assess how well the students learned and to troubleshoot specific deficits in knowledge and skill (Corbett and Mostow 2008) and questions on exams need to be tailored for deeper learning and more discriminating assessments of learning (Scardamalia and Bereiter 1992; Corbett and Mostow 2008; Leacock and Chodorow 2003). More interestingly, problem posing always precedes problem solving and is an important micro-activity that is needed for problem solving (Pintér 2012).

### Related work

PP has been explored by researchers in a number of domains, and dimensions. In Table 1, we present a range of research work which we found during our literature survey.

**Table 1:** Related research on problem posing

[Ref]	Domain (course)	Mode (classroom/lab/online)	Intervention/procedure	Sample/target subject (background and number)	Findings
Gubareva, (1992)	Biochemistry	Classroom lecture	Students were given guidelines of what type of problems to pose before performing PP	Unavailable	Quality of problems improves gradually with more and more PP practice
Graesser and Person (1994)	Research Methodology (RM) and Algebra	Tutorial	PP between tutor and students in a tutoring session	Undergraduates—RM *N* = 27, Seventh graders—Algebra *N* = 13	Evidence—students were able to self-regulate their learning by asking questions when they spot knowledge deficits
Silver et al. (1996)	Mathematics education	Lab experiment	Interleaved PP-problem solving-PP three-level activity on a given context	53 middle school teachers and 28 prospective secondary school teachers	Subjects shown some skills of PP. Subjects posed more problems before problem solving than during or after problem solving. PS influenced the focus in the second PP activity
Silver (1997)	Mathematics education	NA	NA	NA	Discussed that inquiry-oriented mathematics instruction which includes PS and PP tasks and activities can assist students to develop more creative approaches to mathematics
English (1998)	Generic	Experiment	16 sessions (8 weeks) of PP program for improvement of PP skills	Six classes of 8-year-old students (*N* = 154)	Experimental group shown significant improvement in the PP skills—ability to generate their own problems
Cai and Hwang (2002)	Quantitative aptitude	Lab experiment	Three pairs of problem solving (PS) and PP tasks were used in this study	98 US and 155 China-6th grade students	There was a much stronger link between PS and PP for the Chinese sample than there was for the US sample
Mestre (2002)	Physics	Lab experiment	Students were asked to do PP based on the given situation and their prior knowledge	4 undergrads	PP is a powerful assessment tool for probing students’ understanding of physics concepts, as well as their ability to transfer their knowledge to novel contexts
Lavy and Bershadsky (2003)	Mathematics education	Lab experiment	2 workshops with PP activities based on given problem were performed using “what-if-not?” strategy	28 pre-service teachers (second/third year)	Contribution: Categorization of the different kinds of posed problems using the “what-if-not?” strategy
McComas and Abraham (2004)	General	Classroom	NA	NA	Compiled taxonomy of question types. Proposed a 3-step technique to ask effective questions, and 8 factors for asking effective questions to teachers
Profetto-McGrath et al. (2004)	Nursing education	Context-based learning tutorial/seminars	Thirty 90-min seminars were audio taped and analyzed using a Questioning Framework designed for this study	30 nurse educators and their 314 students	Majority of questions posed by tutors and students were framed at the low cognitive level. Recommendations: students and tutors should be trained on how to question
Akay and Boz (2009)	Mathematics education	Classroom	The experimental group was demonstrated with 28 different PP activities	41 prospective science teachers	It reaffirmed that PP (by teachers) should be used in mathematics classes
Toluk-Uçar (2009)	Mathematics education	Classroom	Classroom PP exercise-subjects posed problems on given symbolic situations	95 pre-service primary school teachers	PP had a positive impact on pre-service teachers’ understanding of fractions as well as on their views about what it means to know mathematics
Kar et al. (2010)	Mathematics education	Lab experiment	Prospective teachers (PT) PP-PS tests. Each item in the PS test included patterns in PP tests	76 (PTs)	There was a significant relation between PP and PS
Lavy and Shriki (2010)	Mathematics education	Computer-based environment	Subjects were given guidelines using the “what-if-not?” strategy	25 PTs	PTs perceived that engaging in the inquiry-based activity enhanced both their mathematical and meta-mathematical knowledge
Cankoy and Darbaz (2010)	Mathematics education	Classroom with PP as an instructional strategy	Experimental group has followed a PP-based PS instruction for 10 weeks, whereas the control group has followed a traditional PS instruction	53 third-grade students from an urban elementary school	Experimental group was better than the control group students in terms of understanding the problem even after a 3-month gap between posttest and intervention
Çildir and Sezen (2011)	Physics education	Lab experiment	Study sheets which consisted of 8 PP questions	9 prospective physics teachers-sophomores	High scorers have higher PP skills than those with medium or lower scores; however, no significant difference was observed between those with medium or lower scores in terms of their PS skills
Beal and Cohen (2012)	Mathematics and Science	Online collaborative learning environment (Teach Ourselves)	Pose problems over web-based content-authoring and sharing system	Middle school students, *N* = 224	Evidence—students were able to generate problems on the online platform
Sengül and Katranci (2012)	Mathematics education	Lab experiment	PP related to the “Sets” topic and then qualitative study of their activity	56 sophomore prospective primary mathematics teachers	Subjects had the most difficulty in adjusting the level of the problem posed to the level of the primary education
Arikan et al. (2012)	General	Lab experiment	15 PP-based questions and then qualitative study	8 eleventh graders	The PP activity can also be utilized by teachers as an alternative method of assessment
Pintér (2012)	Mathematics education	Classroom	Initial question, and demo of the “what-if” methods of PP were presented	Small sample of self-selected students in PS course	Improvement in posing problems of “what-if” type
Cai et al. (2013)	Mathematics education	Classroom activity	Combination of PS and PP tasks given to students	390 eleventh graders	Confirmed the validity of PP as a measure of curriculum effect on student learning. Contributed with qualitative analysis rubrics for the questions

The literature survey shows that problem posing has been used as an instructional strategy mostly in the domains of mathematics and prose comprehension. Research in other domains is limited, particularly to physics education, nursing education, and biochemistry. To the best of our knowledge, there is a dearth of research that explores PP as an instructional strategy for teaching–learning of computer science or teaching–learning of engineering domain as a whole. Moreover, no significant research has been found, which talks about student PP skill as an object of instruction. One of the few research that has been found in this direction is about training pre-service teachers on effective question posing. Graesser and Person (1994), Akay and Boz (2009), Lavy and Shriki (2010), and Lavy and Bershadsky (2003) show how some instructions on PP can improve PP skill for some specific type of problems. McComas and Abraham (2004) and Profetto-McGrath et al. (2004) specifically establish need for effective teaching–learning strategies for developing PP skills. Gubareva (1992) talked about how could PP be used in building PP skills in the biochemistry domain. English (1998) and Lavy and Bershadsky (2003) show how some instructions on PP can improve PP skill for some specific type of problems. Beal and Cohen (2012) have demonstrated that mathematics PP skill was improved when the activity was carried out over an online collaborative learning environment.

Mestre (2002), Cai et al. (2013), and Arikan et al. (2012) employ PP as an assessment tool. Toluk-Uçar (2009), Lavy and Shriki (2010), Silver (1997), Cankoy and Darbaz (2010), Gubareva (1992), English (1998), and Pintér (2012) demonstrate how PP can be used as an instructional strategy. Çildir and Sezen (2011) and Silver et al. (1996) talk about the relation between problem posing and problem solving. As far as our exploration of PP as an instructional strategy is concerned, the notion of PP that we are interested in is PP involving the generation of new questions around a given situation, wherein students use the PP activity as a way to unfold new knowledge, around conceptually related seed knowledge, in any given domain. We want that the PP situation should not restrict the posed questions around a specific problem solving task, as in Dillon (1982). However, we want that the PP situation should enable the generation of questions around the scope of a course, and/or a domain. This PP situation is described as a semi-structured PP situation, as opposed to the free and ill-structured PP situations (Stoyanova and Ellerton, 1996). The semi-structured PP situation enables divergent thinking and is driven by students’ intrinsic motivation and therefore positively affects problem posing (Lee and Cho 2007). To the best of our knowledge, there is no existing research that aims at exploring PP as an instructional strategy with this notion in computer science education research.

## Problem posing as an instructional strategy

Literature suggests that PP involves student in the transformation of knowledge and understanding, engages them in constructing knowledge through various processes, and enables them to generate new knowledge through self-exploration (Ghasempour et al. 2013; Beal and Cohen 2012). The PP activities foster a sense of ownership of learning in students by engaging them in metacognitive strategies (Ghasempour et al. 2013). This motivated us to explore PP as a technique through which students can self-direct their learning.

### Designing the PP-based instructional strategy

We employed Design and Development Research (Richey 2014) to develop a QP-based teaching–learning (T-L) strategy to enable student directed learning in classroom settings. Three cycles of Design and Development Research (DDR) has been employed to come up with the current version of the strategy. The developed T-L strategy is known as Student Query Directed Learning (SQDL). The three cycles of DDR are described as follows:

#### The first DDR cycle

**Fig. 1:**
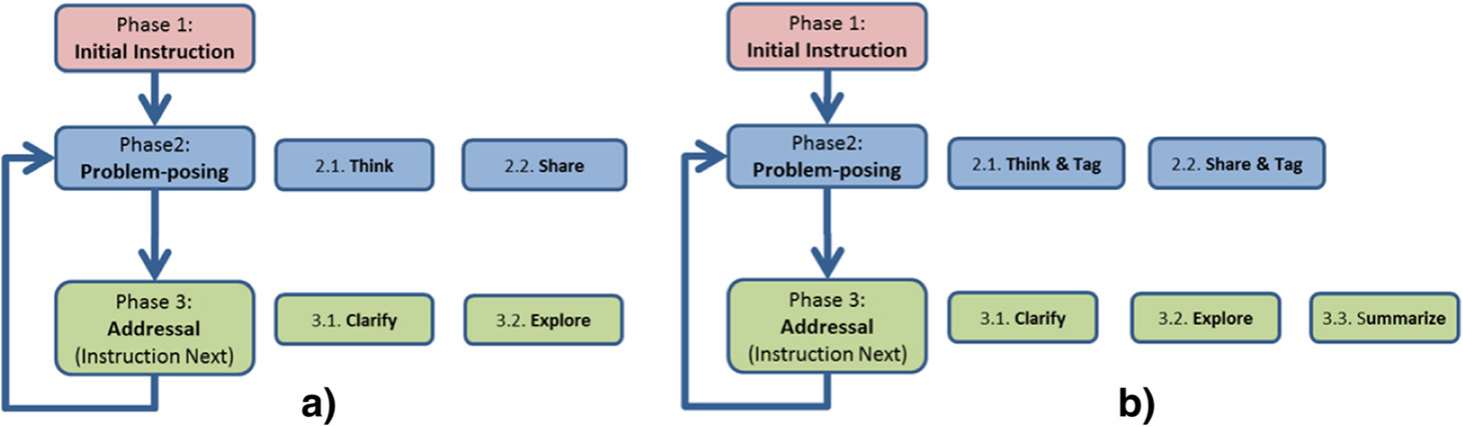
**a** SQDL version 1—the preliminary version. **b** SQDL version 3—the final version

The objective of the first DDR cycle was to come up with a preliminary design of SQDL (Fig. 1a) and investigate if a PP-based activity could be administered with the following constraints: (i) Questions are posed by all students either to address their knowledge deficit, or to construct new knowledge. (ii) Generated questions are reviewed among peers to reduce redundant questions. We started with a straight forward PP-based activity in classroom, where a teacher delivers a small instruction, students write questions during and after the instruction. Students share the questions with each other and return the question slips after removing the repeated questions. After collecting the questions, the teacher answers all the clarification (muddy point) questions and then addresses all the exploratory questions. The first version of the SQDL strategy that satisfies these requirements is comprised of the following three phases of activities:

*Phase 1—initial instruction phase*: The initial instruction phase was used as a semi-structured PP situation (Stoyanova and Ellerton 1996), which was characterized by an initial instruction (seed) by the teacher. The contents of the initial instruction comprise fundamental sub-topics which are essential for the exploration of the complete topic(s). In this paper, we refer to the contents of the initial instruction as “Seed knowledge” or “Seed”. Moreover, this initial instruction explicitly has hints or components, which can encourage exploratory questions among students. The initial instruction was light (less in content), and short (of short time), to ensure that students assimilate (Mayer and Moreno 2003) most of its contents. Students were free to take notes or write questions simultaneously along with attending to the instruction.

*Phase 2—problem posing phase*: In the second phase, students are asked to pose questions around the content they study in the seed. Students are explicitly told that they can generate questions for two purposes—(a) when they want to clarify any muddy point related to the seed or any previous lecture, and (b) when they want to discover more knowledge related to or based on the contents of the seed instruction. We call this activity of question posing as “*think*” sub-phase.

After each student has finished posing questions, they are asked to share their questions among each other (“*Share*” sub-phase). Students are asked to review others’ questions and ensure that the question is not a repetition of their own question. Two students with similar questions were required to disambiguate the question set by removing one of the two similar questions. Students are not asked to discuss the answers with each other, as this would consume enormous amount of time.

*Phase 3—addressal phase (instruction next)*: All the generated questions are collected, and the teacher answered each question one by one. While answering, the teacher is asked to answer “clarification” type questions first (“*Clarify*” sub-phase) and then answer “exploratory” type questions (“*Explore*” sub-phase). By “clarification questions,” we refer to all the questions which require reiteration of the content that has been explicitly been taught in the seed or in any other previous lectures in the course. By “exploratory questions,” we refer to the questions which lead to unfolding or construction of new knowledge. Clarification questions are addressed first because they could be the bottle-neck and pre-requisite for understanding the discussions about exploratory questions. During the “*clarify”* and “*explore”* sub-phases, the instructor has the liberty not to answer irrelevant and out-of-scope questions.

We did a field study based on this preliminary design in an artificial intelligence (AI) course, and identified the required modifications in the strategy, which led to the revised design of the second DDR cycle.

#### The second DDR cycle

Taking inputs from the implementation of the preliminary strategy, we modified the strategy by adding a small activity of “*summarization*” under phase 3. During the “summarization” sub-phase, the teacher summarizes and organizes all the concepts discussed during the “explore” and “clarify” sub-phases. The summarization is essential in order to enable students to make connections between the concepts discussed for a better learning (Fodor and Pylyshyn 1988). We implemented the modified SQDL in a data structures (DS) class. The observations from this implementation suggested further modifications in the SQDL strategy.

#### The third DDR cycle

The modification done in the SQDL strategy for the third DDR cycle was that an activity of *tagging* was added to phase 2 (Fig. 1b), i.e., after posing their own questions (“think” sub-phase) and while reviewing others’ questions (“share” sub-phase), students are asked to tag the questions as “low”, “medium”, and “high” according to their perception of the importance of the questions. This ensured that the sharing activity is not merely a way to avoid the redundant questions, but it made students review the questions even deeper. This modification was in line with the requirement of constructionist learning (Papert 1993), which advocates that learning occurs “especially well when the learner is engaged in constructing something for others to see” (Papert 1993, Patten et al. 2006). In the third (and current) version of the SQDL strategy, it is ensured that students construct new knowledge through question posing, and at the same time, they know that their generated question will be reviewed by others and the answer to the questions will be addressed to or discussed with the whole class.

The field study (field study 1) for the first DDR cycle was executed in artificial intelligence (AI) course, whereas the field studies (field studies 2 and 3, respectively) for the second and the third DDR cycles were administered in data structures classes. There were several types of data collected in each field study, but in this paper, the only data that we discuss is the questions generated by students during problem posing phases of field study 1 and field study 2, as the research focused on how much exploration-based learning took place.

In the next sections, we discuss the final version of SQDL and the results obtained from the qualitative analysis of the questions.

### Defining SQDL—the final version

We define *SQDL* as a question posing-based teaching–learning strategy that enables students to regulate their learning by posing questions. Students’ pose questions based on the contents of an initial lecture (“Seed”) and determine which content/sub-topics that are conceptually related to the seed have to be taught next. After the last DDR cycle in the current version of SQDL, a single iteration of SQDL consists of three phases: (1) *Initial Instruction Phase*, (2) *PP Phase*, (3) *Addressal (or next instruction) phase*. Phase 2 is comprised of two sub-phases: (2.1) *Think and Tag*, (2.2) *Share and tag.* The third phase is comprised of three sub-phases: (3.1) *Clarify*, (3.2) *Explore*, (3.3) *Summarize*.

### Research methodology

In this sub-section we discuss the two implementations of SQDL (field study 1 and field study 2). We delimit our discussion to the collection and analysis of posed questions, with an objective of investigating how much exploration-based learning took place.

#### Implementations (the PP sessions and data collection)

##### Artificial intelligence sessions (field study 1):

We administered two PP sessions in a seventh semester engineering classroom of 35 students in an AI course. The first phase or the seed instruction phase was of 15 min. The topic covered in the seed lecture of the first AI session was “Comparison of Attributes of Intelligence in Utility based, Goal Based, and Simple Reflex agents”. The learning objective for the first session of the seed instruction was “By the end of the seed instruction student should be able to identify differences between simple-reflex, goal-based, and utility-based agents, with respect to the level and attributes of intelligence”.

The topic covered in the seed lecture of the second AI session was “The architecture of learning agents”. Learning objective for this session of the seed instruction was “By the end of the seed instruction student should be able to identify the attributes of intelligence present in the learning agents”. The PP phases in the both sessions continued for 10 min. Students wrote their questions on paper slips and submitted to the TAs. Students were explicitly told about the types (clarification and exploratory) of questions that they could prefer to generate. We collected 25 distinct questions in the first session and 23 distinct questions in the second session.

At the end of the AI session, students were asked to write down their feedback to the open-ended question, “How was today’s lecture different, good, and bad from other traditional lectures?” We received responses from 39 students.

##### Data structure session (field study 2):

Similar to the AI session, we administered a PP session in a 4th semester engineering classroom of 60 students in a DS classroom. The instruction phase was executed for 15 min. Topics covered in the seed lecture were “Node Structure” and “Linking two nodes”. The learning objective of the seed instruction was “By the end of the seed instruction, student should be able to define, declare, construct, and access their own nodes and linkages between nodes using Java.” The PP phase continued for 10–15 min. Students were told to write their questions on paper slips, review the questions from their peers to remove the redundant questions, and submit the final question slips to the TAs. After discarding the irrelevant and remaining redundant questions, we were left with a corpus of 56 distinct questions.

#### Data analysis

##### Grounded theory-based qualitative analysis:

We have collected a total of 104 student questions from the two PP sessions. We first conducted an in-depth study of these question statements to find out what strategies students use to pose questions in the given semi-structured PP situation. We employed a grounded theory-based inductive qualitative research methodology. After the completion of the analysis, we found the answer to the more refined research question, “How do students use their prior knowledge/experience, and the knowledge from “seed” to generate a new question?” In this paper, we are not reporting the detailed analysis procedures and output, as it has been communicated for publication elsewhere. The result of the analysis was eight PP strategies that explain how students used prior, and the seed knowledge to come up with new questions.

##### Content analysis:

We further qualitatively analyzed each question to extract the knowledge type of the prior knowledge used to generate the question, knowledge type of the unfolded knowledge for any question, concept (topic/sub-topic) unfolded by any question.

In this paper, we present a descriptive analysis of different PP strategies evident for the question set, the knowledge types of the information requested by the question set, and the amount of knowledge unfolded using PP. The next section contains the analysis results of the study.

##### Open-ended feedback from students:

To analyze the open-ended responses from all students, we performed a content analysis of the text obtained from their feedback notes. We coded each response to answer three questions: (1) What are the advantages of the PP-based SQDL activity? (2) What are the disadvantages of the activity? (3) Reason behind advantage and/or disadvantages?

### Results (PP as an instructional strategy)

#### PP strategies

PP strategies emerged out of grounded theory-based inductive qualitative analysis of 104 questions are described in Table 2. It should be noted that for a complete inductive model, further work, with more data, is needed, and therefore, the evolved strategies may be further refined in future research. We have used the Bloom’s 2-D taxonomy of knowledge type (Anderson et al. 2001) and identified different types of knowledge that students unfold. An account of type of knowledge unfolded in all the seven sub-strategies are given in Table 3. In this case, the frequencies do not sum to 1 because there were few questions which fell in more than one strategy. We see that out of four knowledge types defined in the Bloom’s 2-D taxonomy (Anderson et al. 2001), metacognitive knowledge type could not be unfolded. We also see that except ‘*Clarify’*, all other seven strategies lead to knowledge unfolding. We are not reporting the analysis procedure, as it has been communicated for publication elsewhere.

**Table 2:** PP strategies evolved from the grounded theory-based qualitative analysis of questions

Strategies	Definition	Example
Apply	The seed knowledge is employed to create some “known application” from prior knowledge. Explicit identification of prior known application is mandatory in this strategy. Applications are identified either from: 1) the same domain, or 2a) different academic domain, or 2b) real life.	*“*Creating *social network graph, is it possible?”,* Here application (“*social network graph*”) comes from real life experiences.
Organize	This strategy aims at unfolding variants of the seed knowledge by organizing multiple instances of the seed concept to obtain some structural arrangement (which comes from prior experience).	*“Cyclic list of nodes possible?”* Here multiple instances of the concept “*node”* (from seed knowledge), i.e., large number of *nodes* are proposed to be organized in a cyclic manner to unfold a variant of the seed (i.e., *circular linked list*).
Probe	Prior knowledge is used as a basis to make a richer inquiry into the seed and used to add more understanding of the seed. Here prior knowledge is *NOT* the prior known application, as in *Apply*. Associations between prior knowledge and seed knowledge are performed so as to use prior knowledge as a basis to make a richer enquiry into the seed knowledge.	Example: *“address (next) is relative or direct?”* Here concepts from prior knowledge (“*relative/direct addressing”)* has been used to make a richer understanding of the construct “*next*”, which is a part of seed.
Compare	The questioning strategy is to make associations between prior knowledge and seed knowledge such that prior knowledge is compared or contrasted with the concepts in the seed knowledge.	Example: *“chain of nodes vs. array?”* In this question the prior knowledge (“*array”*) is contrasted with seed concept (chain of *“nodes”)*.
Connect	In this strategy, student associates the seed knowledge to some prior knowledge, from same domain, from other domains, or from real life. Making analogy between some prior knowledge with seed knowledge is included in this strategy. Contrasting or comparing the seed with some prior knowledge does *NOT* come under this strategy.	Example: “*Can we use neural network and fuzzy logic to create an agent?”* In this question. the prior concept of “*neural network* and *fuzzy logic”* is connected with the context of seed knowledge (“*an agent”)*.
Vary	In this strategy, the objective of the question is to modify/ vary the component(s), attribute(s), or part(s) of the seed to unfold the variants of the seed concepts. These questions may or may not give rise to some application of the seed, but applications are *NOT* explicitly identified.	*“In addition to next have previous node?”* In this question, instead of having just one pointer/reference to another node, the idea of having two pointer/ reference variables in the node structure, is proposed. In this way a variant of “*singly linking”* (i.e., a “*doubly linking”*) is unfolded.
Implement	The questions generated using this strategy show that students think about how some operation/procedure, can be performed on the seed knowledge to achieve a goal state related to the seed. It should be noted that prior knowledge, which is in the form of operation/procedure, are explicitly evident from the question statement.	*“How to perform inheritance from a node possible to give “multinodes*”?” Here the operation inheritance has been explicitly identified, and question is about how to implement that operation on the seed concept (“*nodes”*).
Clarify	The analyses revealed that students ask question to clarify their muddy points. All the questions which needed reiteration of the content that has been explicitly been taught in the seed or in any other previous lecture in the course are categorized to follow clarification strategy. Hence clarification questions do not unfold any new knowledge.	*“What is the use of ‘this’ method?”* The use of “this” operator was explicitly taught in the seed.

**Table 3:** Frequency of applications of different PP strategies and related knowledge types

Strategies	Knowledge type	Knowledge type
	(Prior knowledge)	(Unfolded knowledge)
	(*N* = 104)	(*N* = 104)
Apply	Conceptual (0.14), Procedural (0.01)	Conceptual (0.14)
Organize	Conceptual (0.08), Meta-Cog(0.01)	Conceptual (0.05), Procedural (0.02), Factual (0.01)
Probe	Conceptual (0.13), Procedural (0.02),	Conceptual (0.07), Factual (0.07)
	Meta-cog (0.11)	Procedural (0.02),
Compare	Conceptual (0.05), Procedural (0.01), meta-cog (0.01)	Conceptual (0.06), Procedural (0.01)
Connect	Conceptual (0.05), Factual (0.02), Meta-cog (0.03)	Conceptual (0.05), Factual (0.04), Procedural (0.01)
Vary	Conceptual (0.13), Procedural (0.01), Factual (0.01)	Conceptual (0.01), Procedural (0.08), Factual (0.01)
Implement	Conceptual (4), Factual (0 + 11), Procedural (2 + 3 + 1), Meta-cog (0 + 1 + 1)	Conceptual (0.02), Procedural (0.03)

#### Knowledge unfolded

**Fig. 2:**
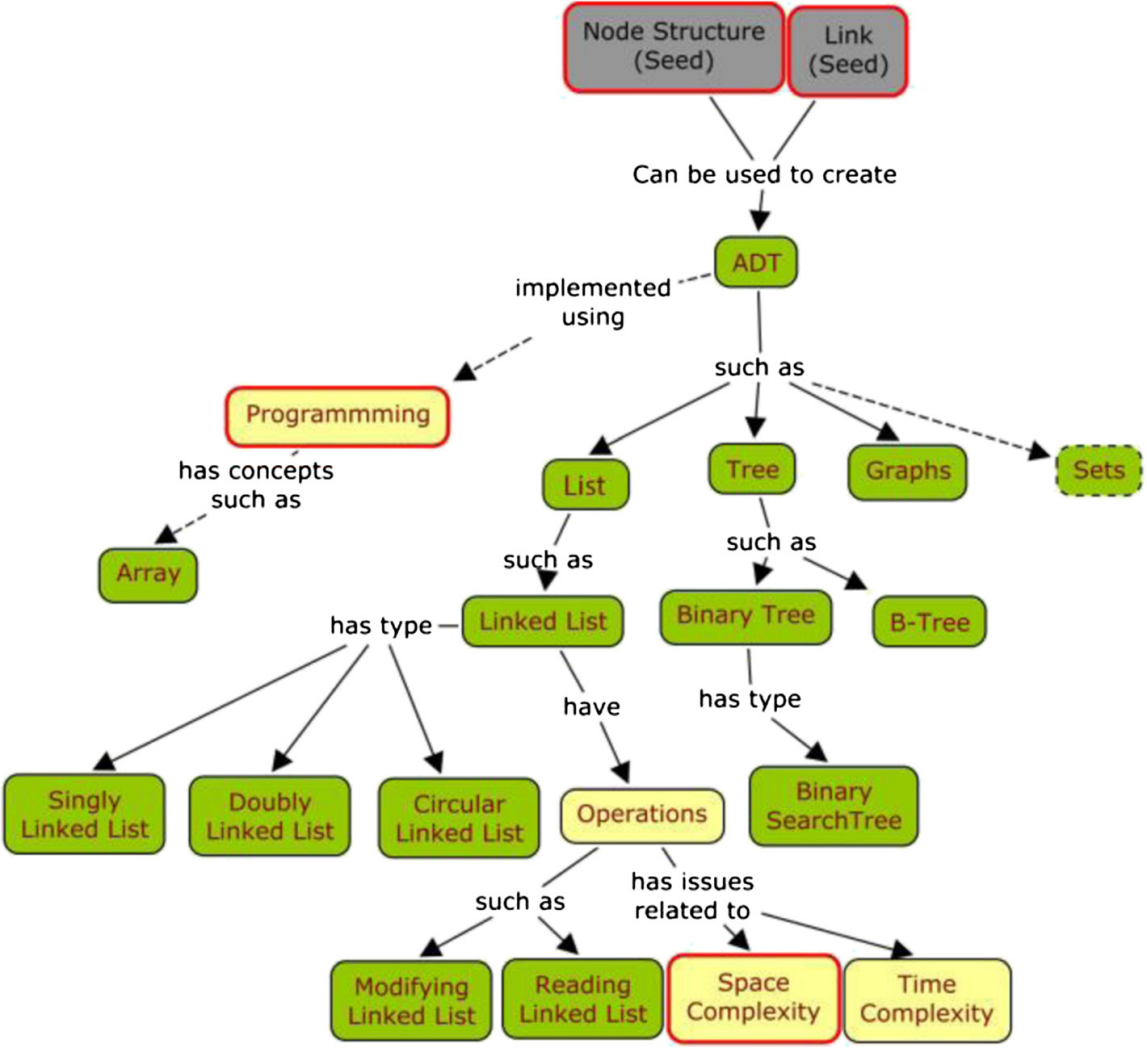
Concepts unfolded in data structures PP session

We found a range of topics/sub-topics from the traditional syllabus has been unfolded by the PP activities. The concept map of the concepts unfolded in the DS session is shown in Fig. 2. The concept map was created in consultation with the course instructor. The nodes in the concept map are the different concepts requested by the generated question.

The grey nodes represent the concepts which were taught in the instruction phase (i.e., seed concepts), while the red border around a node denotes that there were some clarification questions generated related to that particular concept. The green nodes show the concepts which were unfolded, i.e., they were not taught to students before. The green node with a dotted border is an unfolded concept which is out of the scope of traditional DS syllabus. The concepts denoted by the yellow nodes are the prior knowledge within the domain which was used during PP.

#### Student’s open-ended feedback

We found that all of the 39 responses suggest that the activity was helpful in learning and creating interest. Students predominantly perceived that the activity was helpful to learning due to the following reasons: (a) The activity helped them to clarify their muddy points and learning basic details. (b) Due to the activity, students came across critical questions. (c) The activity covers all necessary topics. (d) It was better to explore topics more from students point of view. (e) It removed fear and hesitation of participating in the class, and increased active learning. The disadvantage of the activity as perceived by the students was that the activity was very much time-consuming. It would be interesting to study in our future research how much time does the traditional lecture require as compared to the time required by the SQDL approach to cover the same set of topics.

## Problem posing as an assessment tool

We conducted an in situ field study in the CS101 course. The main objective of the study was to investigate the effect of a 2-week scratch intervention on students’ learning and transitioning to C++. We aided the study with a PP-based nontraditional assessment tool. We designed a PP-based assessment activity, to investigate the learning of computational thinking concepts of introductory programming. The research questions that this study answered were:

*RQ1: How can student-generated questions be used to assess the learning of Computer Programming concepts?*

*RQ2: How does the quality of question(s) generated by a student relate to the score achieved by him/her in the traditional assessment?*

The PP situation of this activity was completely different than that described in the “Problem posing as an instructional strategy” section. Here the “Seed” knowledge was considered to be the 4-week-long (total 12 h) instruction. Moreover, the purpose of this PP activity was to generate questions to assess other students, whereas the purpose of question posing in the SQDL (“Problem posing as an instructional strategy” section) was to clarify or explore knowledge to improve learning.

### Research methodology

#### The PP activity implementation

**Fig. 3:**
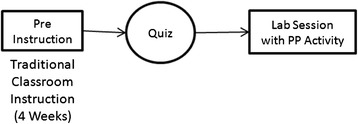
Implementation of PP as an assessment tool

After teaching CS1 for 4 weeks using Scratch and C++ as the programming languages, we conducted a traditional assessment in the form of a quiz. In the fifth week, during the lab sessions, each student was asked to generate two practice questions for the coming mid-term exam on which other students could work (Fig. 3). Since PP was a novel activity for students, this novelty could have obstructed smooth responses from students. Literature (Williams et al. 2000; McDowell et al. 2002) suggests that collaboration is an effective pedagogical tool for teaching introductory programming. In the case of learning through pair programming, students produced better programs and completed the course at higher rates. This motivated us to make students to generate programming questions in pair. Therefore, we implemented a “collaborative” PP activity in which two students collaborated as a team to generate questions. Each pair was asked to generate two questions pertaining to the topics covered so far. They were free to set either a programming question or a conceptual question and had to submit detailed answers to their generated questions. Students were given motivation that the 18 best questions from each lab-batch would be selected as the practice questions for the next lab-batch, and questions could be selected for the mid-semester question paper. Students were given only one open-ended guideline “The questions should be challenging but should not be too difficult for the students in the next batch to complete in the lab”. The time given to generate two questions was 45 min, but for many students, the time was extended up to an hour. Students submitted their generated questions over Moodle (Dougiamas and Taylor 2003), the learning management system used in the course.

A team of TAs was assigned to talk to students and motivate them to brainstorm and generate questions that may lead to deeper application of the concepts taught in the class. There were 90 students per lab session, and there was one TA per 10 students. TAs were told to intervene whenever they found any student stuck in the activity, or sitting idle for long time, or busy doing some out-of-context work. It was the responsibility of a senior TA to coordinate with junior TAs to manage all the logistics in the lab session.

#### Data analysis

The generated questions were analyzed qualitatively to answer our research questions. We analyzed the questions based on five different qualitative analysis themes, viz., creativity of the problem poser, difficulty of the problem, cognitive level of the problem, problem type, programming concepts (Table 4).

**Table 4:** Parameters for qualitative analysis of problem posed in programming domain

Parameters	Creativity of the problem poser	Difficulty of the problem	Bloom’s level	Problem type	Programming concepts (can take one or more values)
Values	Low, medium, high	Low, medium, high	Recall, understand, apply, analyze, evaluate, create	Write a program, debugging, predict the output, theoretical (subjective)	Sequence, loops, parallelism (threads), events, conditionals, operators, data (non-array), data arrays

We designed qualitative rubrics to define different levels of creativity and difficulty of generated problems. This rubric (given in Table 5) was prepared in consultation with three Educational Technology researchers who had at least 17 years of experience in computer programming. To analyze which programming concept is targeted by any problem, we used the list of Computational Thinking Concepts (CTCs) given by Brennan and Resnick (2012). We found that each question contained one or more CTCs. Cognitive levels of the problems were assigned as per revised Bloom’s Taxonomy (Anderson et al 2001), and we also analyzed whether the problem type is of write a program, debugging, predict the output, or theoretical (subjective).

**Table 5:** Rubrics for analyzing creativity and difficulty levels

	Low	Medium	High
Creativity of the problem poser	The context addressed in the problem is same as textbook programming problems e.g. “*Check if a number is prime”*. The use of constructs is conventional.	Prior knowledge used in the problem comes from courses experienced in school level.	Attempt of a new context (prior knowledge used in the problem comes from the real-world experiences) and innovative use of constructs.
Difficulty of the problem	Problems with well-understood logic and straightforward solution.	Problems with some amount of logical challenge and do not have a straightforward solution.	Problems which are highly logically challenging and have no straight forward solution.

To answer RQ2, we operationalized the quality of questions using difficulty levels of the questions. Then we explicated the pattern between the difficulty level and the stratified (low, medium, high) scores of the fourth week quiz using stratified attribute tracking diagrams (Majumdar and Iyer 2014).

### Results (PP as an instructional strategy)

#### Learning of programming concepts

We find that *Operator, Data,* and *Sequences* were the prominent CTCs targeted by most of the generated questions, while almost 70 % of the generated questions requested the knowledge of *loops* (Table 6).

**Table 6:** Frequencies of questions exhibiting different CTCs

Computational Thinking Concepts (CTC)	Sequence	Loops	Parallelism (threads)	Events	Conditionals	Operators	Data
Percent of questions requesting any (CTC)	91.82	69.81	8.18	8.81	63.52	94.34	96.23

#### Quality of questions (difficulty levels and creativity)

Frequency distributions of questions with different difficulty and creativity levels are shown in Tables 7 and 8, respectively. It should be noted that these frequency distributions tell us about the performance of the class as a whole and not the individual students.

**Table 7:** Difficulty level distribution of questions

Difficulty levels	Percent of questions of any difficulty level
High	10.06
Medium	50.94
Low	38.99

**Table 8:** Creativity level distribution of questions

Creativity levels	Percent of questions of any difficulty level
High	10.06
Medium	50
Low	40

#### Relation between the traditional assessment score and nontraditional assessment tools

**Fig. 4:**
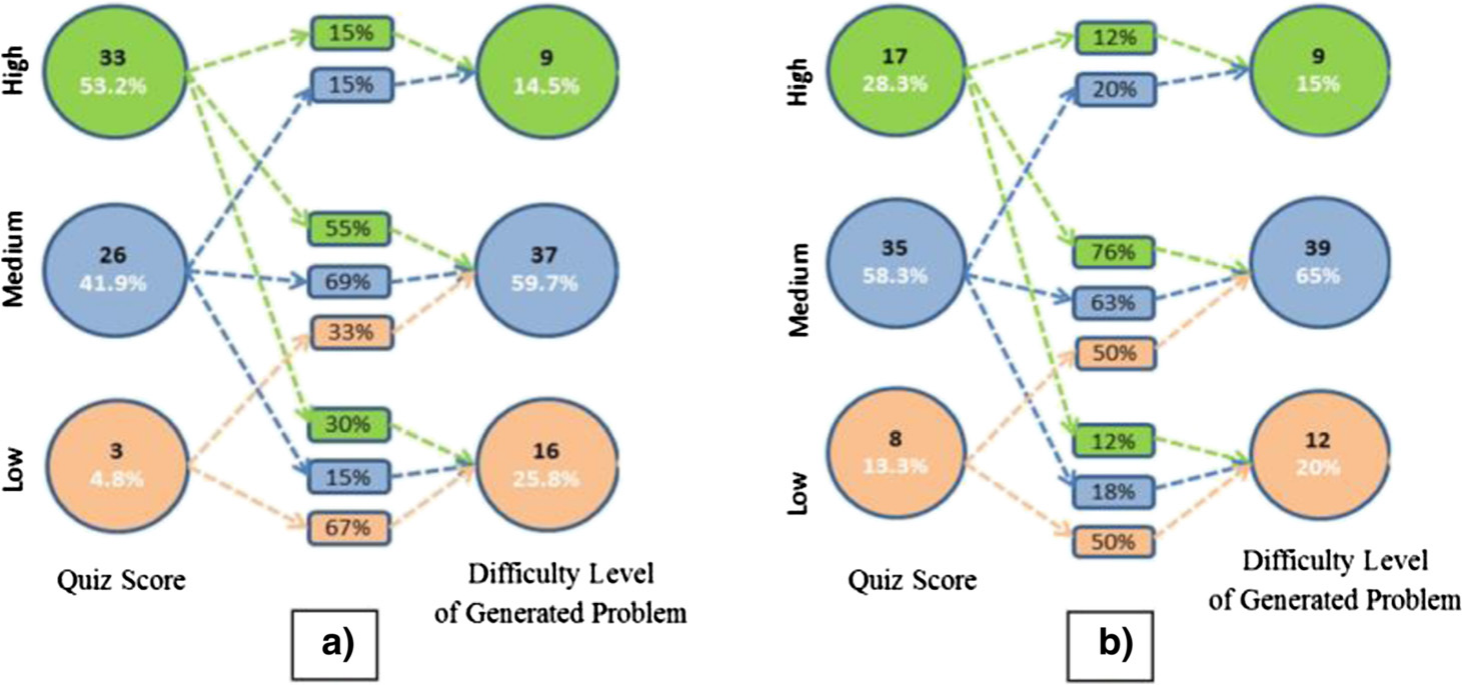
**a** Advanced learners. **b** Novice learners

Figure 4a, b shows the transition patterns of performance of students in traditional vs nontraditional assessments. Figure 4a shows the pattern for advance learners, and Fig. 4b shows pattern for novices.

We see that for novices, the higher the score in the quiz, the lesser is the probability of generating a low difficulty question. Probability of generating medium-level difficulty questions by both high- and medium-level quiz performers is evident in both novice and advance cases. Interestingly, high probability of generating low difficulty questions by high pretest performers is evident in the case of advance learners only, this shows that the difficulty level can be used to assess the learning of novices, but not advanced learners.

## Discussion and conclusion

We designed two kinds of PP activities with two different PP situations. The nature of PP situations varied depending on the purpose of PP. The first PP situation aimed at exploring the potential of problem posing as a tool to facilitate exploration-based learning. We had employed DDR and developed SQDL as a PP-based instructional strategy. We implemented SQDL in AI and DS classes, and collected the questions posed by students during the SQDL sessions. The inductive qualitative analysis of the posed questions revealed eight different strategies by which students use the seed knowledge and their prior knowledge to generate new questions. Out of the eight strategies, seven strategies lead to knowledge unfolding and one of them leads to clarification of muddy points. It should be noted that future research may lead to further refinement of these eight strategies. When we analyzed the questions for different contents (topics/sub-topics) that they request and hence unfold, we found that students were able to unfold a large number of topics in a single iteration of SQDL. The benefits of PP activity are found to be twofold: (a) potential of addressing muddy points through the generation of clarification questions and (b) knowledge unfolding capability through generation of exploratory questions. As far as the extent of knowledge unfolding is concerned, it was evident that there were large numbers of concepts unfolded during SQDL sessions; still there is no metric to determine what should be called as “adequate” or “acceptable” coverage. This could be an interesting future research objective. The responses of the students to the open-ended feedback question “How was today’s activity helpful?” in the class confirm the above. Some of the responses are given below:

- “…Helpful for doubts”
- “…Innovative way of learning…. doubts without being scared”,
- “Through today’s activity… I can explore more… can
- “find new ideas how far we can go with the subject”,
- “…Good way of getting knowledge…”
- “It helped in explore topics more from student point of view and hence improved learning…”

SQDL is helpful in student-driven unfolding of course contents which are conceptually related to the seed instruction. However, we do not expect students to ask questions and unfold topics which are conceptually unrelated to the seed concepts. Therefore, in addition to AI and DS, SQDL is suitable for all domains which has a large number of conceptually related topics. The types and distribution PP strategies employed may vary according to the nature of different domains. We believe that there exists potentially interesting research objective to investigate the variations in nature of questions posed across different domains.

The second PP situation (PPE activity) was designed to explore the potential of problem posing as an assessment tool. We found that PP can be used to assess the learning of computational thinking concepts by students in the CS1 course. In the PPE activity, students generate questions and they also provide solutions/answers to them. This ensures that the concepts which are required to answer a question are understood by the students. We aggregated all these concepts that emerged from the generated questions and determined the frequency distribution of various concepts learned by the students. It should be noted that we did not assess the learning of any individual student on the topics around which (s)he has not generated questions. Though, PPE can be used to assess the learning of different concepts by the class, as a whole. We also attempted to study the relation of “understanding of programming” (operationalized by the quiz scores) with question quality (operationalized by the “difficulty level” of the questions). We found that for novice learners, the higher the score in the quiz, the lesser was the probability of generating low difficulty question. Interestingly, in the case of advance learners, we found a high probability of generating low difficulty questions by high quiz performers. This shows that the difficulty level can be used to assess the learning of novices, but not of advanced learners. Moreover, it is also possible that in addition to “understanding of programming” the “difficulty level” of the generated question might be affected by other factors. Although the results in the paper show some relation between the traditional assessment scores and PPE-based assessment, we do not claim any statistical correlation.

With content analysis of questions for the concepts that any question relates to, PPE can be used in other domains for assessing the conceptual understanding. As far as the difficulty level and other quality parameters are concerned, different domains may need different rubrics for analysis. The use of PPE as an assessment tool shows that different qualitative aspects of questions can reveal a lot about different aspects of learning, and other cognitive and affective parameters. For example, the account of creativity shows how much students are able to relate the concepts to their prior (real-world or academic) experiences. More of these aspects are to be identified to make PP useful for assessing a wide range of objectives.
